# Maintenance N-acetyl cysteine treatment for bipolar disorder: A double-blind randomized placebo controlled trial

**DOI:** 10.1186/1741-7015-10-91

**Published:** 2012-08-14

**Authors:** Michael Berk, Olivia M Dean, Sue M Cotton, Clarissa S Gama, Flavio Kapczinski, Brisa Fernandes, Kristy Kohlmann, Susan Jeavons, Karen Hewitt, Kirsteen Moss, Christine Allwang, Ian Schapkaitz, Heidi Cobb, Ashley I Bush, Seetal Dodd, Gin S Malhi

**Affiliations:** 1 Deakin University, School of Medicine, Barwon Health, P.O. Box 291, Geelong, 3220, Australia; 2 Orygen Youth Health Research Centre, 35 Poplar Rd, Parkville, 3052, Australia; 3 Centre of Youth Mental Health, University of Melbourne, 35 Poplar Rd, Parkville, 3052, Australia; 4 Mental Health Research Institute, University of Melbourne, Kenneth Myer Building, 30 Royal Parade, 3052, Parkville, Australia; 5 University of Melbourne, Department of Psychiatry, Level 1 North, Main Block, Royal Melbourne Hospital, Parkville, 3052, Australia; 6 Laboratory of Molecular Psychiatry, INCT for Translational Medicine, CNPq. Hospital de Clínicas de Porto Alegre, Universidade Federal do Rio Grande do Sul, Porto Alegre, 90010-000, Brazil; 7 Post-graduate Program in Biological Sciences: Biochemistry, Federal University of Rio Grande do Sul - UFRGS -, Porto Alegre, 90035-000, Brazil; 8 Laboratory of Calcium Binding Proteins in the Central Nervous System, Department of Biochemistry, Federal University of Rio Grande do Sul - UFRGS -, Porto Alegre, 90035-000, Brazil; 9 Discipline of Psychiatry, Sydney Medical School, University of Sydney, Sydney, 2006, Australia; 10 CADE Clinic, Department of Psychiatry, Level 5 Building 36 Royal North Shore Hospital, St. Leonards, 2065, Australia; 11 Department of Psychosomatic Medicine and Psychotherapy, Klinikum rechts der Isar der TU, München, Ismaninger Str. 22, 81675, Münich, Germany

**Keywords:** N-acetyl cysteine, depression, bipolar disorder, maintenance, mania, oxidative

## Abstract

**Background:**

N-acetyl cysteine (NAC) is a glutathione precursor that has been shown to have antidepressant efficacy in a placebo-controlled trial. The current study aimed to investigate the maintenance effects of NAC following eight weeks of open-label treatment for bipolar disorder.

**Method:**

The efficacy of a double blind randomized placebo controlled trial of 2 g/day NAC as adjunct maintenance treatment for bipolar disorder was examined. Participants (n = 149) had a Montgomery Asberg Depression Rating Score of ≥12 at trial entry and, after eight weeks of open-label NAC treatment, were randomized to adjunctive NAC or placebo, in addition to treatment as usual. Participants (primarily outpatients) were recruited through public and private services and through newspaper advertisements. Time to intervention for a mood episode was the primary endpoint of the study, and changes in mood symptoms, functionality and quality of life measures were secondary outcomes.

**Results:**

There was a substantial decrease in symptoms during the eight-week open-label NAC treatment phase. During the subsequent double-blind phase, there was minimal further change in outcome measures with scores remaining low. Consequently, from this low plateau, between-group differences did not emerge on recurrence, clinical functioning or quality of life measures.

**Conclusions:**

There were no significant between-group differences in recurrence or symptomatic outcomes during the maintenance phase of the trial; however, these findings may be confounded by limitations.

**Trial Registration:**

The trial was registered with the Australian New Zealand Clinical Trials Registry (ACTRN12607000074493).

## Background

Bipolar disorder is a recurrent illness with the vast majority of individuals experiencing relapses throughout their lives. The prevention of further episodes is of critical importance to individuals with the disorder, as recurrent episodes can result in hospitalization, suicide and loss of functionality. There appears to be an active process of neuroprogression associated with acute episodes of illness [1]. Maintenance of well-being is, therefore, of paramount importance [2]. Existing agents are imperfect, as many have limitations in terms of either efficacy or tolerability for long-term treatment. Lithium is the mainstay of prophylaxis in bipolar disorder although it is more effective in preventing manic relapses than depression [3] though recent data suggest that it is more effective in relapse prevention than valproate [4]. Interestingly, lithium is more effective in preventing manic relapses as opposed to depression whereas lamotrigine is more effective in the prevention of depressive episodes [3]. Atypical antipsychotics also appear to have maintenance properties, although all except quetiapine are less effective in depression than mania. Given the limitations of these agents, polypharmacy is the routine rather than the exception [5] and most have significant tolerability issues that require routine safety monitoring [6]. In this context there are preliminary data from preclinical studies that N-acetyl cysteine (NAC) might prevent lithium-induced renal dysfunction in animal models. This makes it an attractive adjunct therapy both because of its potential clinical benefits and the reduction of iatrogenic adverse effects [7].

This is particularly interesting because it is consistent with evidence of dysregulated redox biology in bipolar disorder. Data supporting this comes from five main areas; i) evidence of dysregulated oxidative defenses, ii) effects of oxidative stress on cellular constituents (particularly lipids, proteins and nuclear and mitochondrial DNA), iii) concordant structural evidence of neuroprogressive processes, iv) studies showing that established bipolar disorder treatments have significant influences on oxidative processes, and v) association studies of polymorphisms of key genes in the glutathione pathway [8]. In particular, glutathione, which is the principal endogenous antioxidant in the brain, is vulnerable to depletion, and is substantially reduced in bipolar disorder [9]. NAC provides L-cysteine, the rate limiting factor in glutathione synthesis, and thereby increases central and peripheral glutathione [10]. Additionally, NAC modulates glutamate, has anti-inflammatory properties and enhances neurogenesis and mitochondrial function [11].

Given this context, the aim of this study was to investigate the efficacy of adjunctive NAC, in addition to treatment as usual, in the maintenance treatment of bipolar depression in a double-blind randomized multi-center placebo-controlled trial. Time to intervention for a mood episode was the primary outcome measure. Secondary outcome measures included changes in mood symptoms, functioning and quality of life (QoL). It was hypothesized that NAC would reduce the recurrence of episodes in the maintenance phase of the disorder.

## Methods

This maintenance study included participants screened for the presence of depression at trial entry (beginning of the open-label phase) [12]. All participants received 2 grams of NAC (1 gram twice daily) for eight weeks and were subsequently randomized to continued NAC treatment or placebo in a double blind design for a further 24 weeks. The assessment schedule is shown in Additional File 1. In addition to the clinical interviews, some participants provided blood samples for peripheral analysis of oxidative stress markers and a sub-group was involved in a magnetic resonance spectroscopy study (data presented elsewhere). In order to reduce enrichment bias, response to NAC in the open label phase was not an inclusion criterion, and all participants proceeded to the randomized phase (week 8 to week 32). Methodological details and data from the open-label phase of the study are presented elsewhere [13]. All participants remained on treatment as usual for the duration of the trial. This included any pharmacological or psychological intervention (stable for at least one month as per the inclusion criteria). In order to capture the diversity of treatment settings and enhance generalizability, potential participants were recruited through a variety of avenues, including the participants' case clinicians, newspaper advertisement, flyers in public areas (including flyers placed at shopping centers and pathology collection centers) and web-based advertisements on bipolar disorder-relevant websites, as well as referral from private clinicians including family physicians and specialists. The trial was approved by relevant research and ethics committees (Barwon Health, Bendigo Health and the University of Melbourne, in Victoria, Australia; Royal North Shore Hospital, Sydney, Australia, and Porto Allegre, Brazil) and was conducted in accordance with the Helsinki Declaration as revised in 1989. A preliminary interview was conducted with potential participants to obtain written informed consent and assess inclusion and exclusion criteria, following which the trial proper commenced.

Individuals who had given written consent were assigned, using computer-generated block randomization (in blocks of four) [14], to treatment with NAC or placebo in addition to treatment as usual, in a double-blind fashion. The nature and dose of the primary therapy was monitored. The person generating the randomization schedule was not involved in any aspect of participant interview or data analysis. The investigators, clinicians and statisticians were blind to treatment allocation. The study was registered with the Australian and New Zealand Clinical Trials Registry (registration # 12607000074493). The trial was completed between 2007 and 2010.

NAC was acquired from Zambon Fine Chemicals, Bresso, Italy. Purity was 99.8% as determined by high performance liquid chromatography. Encapsulation of both the NAC and the identical placebo capsules was done by DFC Thompson, Sydney, Australia. Study medication was sealed in identical bottles, labeled as trial medication, and both dispensed and returned by the pharmacy, so that the investigators were not exposed to the contents of the bottles. It is important to note that NAC has a characteristic odor and so to reduce the risk of unblinding on transition to the double blind phase, placebo capsules were dusted with microgram amounts of NAC to capture the distinct odor. Participants additionally were seen separately, minimizing the opportunity to compare experiences.

### Inclusion and Exclusion Criteria

To be eligible for the trial, participants were required to meet DSM-IV criteria for bipolar I-, bipolar II- or bipolar NOS-disorder, to have current symptoms of depression, with a Montgomery Asberg Depression Rating Scale (MADRS) score of ≥12 at entry into the study, have the capacity to consent to the study and comply with study procedures, be using effective contraception if female, sexually active and of childbearing age and have been on stable therapy for at least four weeks prior to randomization. Participants were not, however, required to be taking medication at the time of recruitment. Exclusion criteria included individuals with a known or suspected clinically relevant acute systemic medical disorder, elderly people with respiratory insufficiency, women who were pregnant or lactating, participants taking more than 500 mg of NAC/day, 200 ug of selenium/day or 500 IU of Vitamin E/day, or who have had an anaphylactic reaction to NAC, or any component of the preparation, or who were assessed as being unable to comply with either the requirements of informed consent or the treatment protocol. Withdrawal criteria included individuals who ceased taking their trial medication for seven consecutive days or who ceased effective contraception or became pregnant. Dose changes to existing medications (either increases or decreases in dose), or addition or removal of an agent were noted and participants were allowed to continue with the trial. Additionally, participants were withdrawn from the study if they withdrew consent, or developed adverse events that were deemed to require withdrawal from the study.

### Measurements

The participants were assessed at the commencement of the open-label phase using the Mini International Neuropsychiatric Interview Plus (MINI-Plus) [15]. Time to any intervention for mood symptoms was the primary outcome measure of the study. The specified interventions included initiation of a new medication, psychotherapy, hospitalization or electroconvulsive therapy, initiation of emergency/unscheduled medical contacts for mood symptoms or discontinuation or dose adjustment of a current agent. To be considered an 'event', participants had to experience one of the interventions directly in relation to the presence of a new mood episode. Meeting time to intervention criteria was not in itself a reason for trial discontinuation, and such individuals, who consented, continued to be monitored, although only the first such event was used for analysis of the primary outcome. Change in the clinical status of the participants was further assessed using the MADRS [16], Bipolar Depression Rating Scale (BDRS) [17], Young Mania Rating Scale (YMRS) [18], Clinical Global Impression (CGI) improvement and severity scales [19], CGI modified for substance use [20] and bipolar disorder (CGI BP) [21], Patient Global Impression (PGI) [22], Global Assessment of Functioning Scale (GAF) [23], Social and Occupational Functioning Assessment Scale (SOFAS) [24], Streamed Longitudinal Interview Clinical Evaluation from the Longitudinal Interview Follow-up Evaluation (SLICE/LIFE) [25] and Range of Impaired Functioning Tool (LIFE RIFT) [26], and the Quality of Life Enjoyment and Satisfaction Questionnaire (Q-LES-Q) [27]. Adherence was monitored by an independent pharmacist, using pill counts of returned clinical trial material. Rating scales were repeated every two weeks for the first four weeks, thereafter monthly or on the day of study termination if the participant withdrew prior to the final scheduled visit [see Additional File 1]. Adverse events were tabulated.

### Statistical analysis

The last visit of the open-label phase (week 8) served as the baseline for the maintenance phase of the trial and the endpoint corresponded to the assessment at week 32. All randomized participants who had at least one post-baseline assessment were included in the analysis. Analysis was performed by a consultant statistician, who was blind to treatment assignment, using IBM® SPSS® Statistics Version 19 on a cleaned and locked database.

All analyses were conducted in accordance with the International Conference on Harmonization E9 statistical principles (International Conference on Harmonization, [10]. Assuming a correlation of post-treatment scores with baseline measurements of 0.7 and an effect of the dosage such that experimental (usual treatment and NAC) group differs from controls (usual treatment and placebo) by 0.75 standard deviations, power was maintained above 90% with 75 subjects in each group. Differences in the two groups in baseline demographic and clinical characteristics (week eight at randomization) were examined using independent samples t-tests and chi-square analysis (χ^2^). These inferential statistics were also used to compare participants who were included/excluded from the maintenance phase of the trial and who completed/discontinued the intervention.

Fisher's exact test was used to determine differences between the two groups with respect to the frequency of interventions for mood episodes. Kaplan Meier estimates and the Mantel-Cox log-rank test (χ^2^) were used to evaluate differences in time to intervention for a mood episode between the NAC and placebo groups (primary outcome). Time to depressive episode was also analyzed using these techniques (secondary outcome).

The analyses of continuous secondary outcome measures involved the use of a likelihood based mixed-effects model repeated measures approach (MMRM). The MMRM model included the fixed, categorical effects of group, visit, and group-by-visit interaction. The MMRM includes all available data at each time point [28] and is the favored approach for analysis of data from clinical trials in psychiatry [29]. The Toeplitiz covariance structure was used to model the relations between observations on different occasions. Planned comparisons using MMRM were conducted to examine group differences in mean change on the outcome measures from baseline (week 8) to endpoint (week 32). All tests of treatment effects were conducted using a two-sided alpha level of 0.05.

## Results

### Sample characteristics

**Figure 1:**
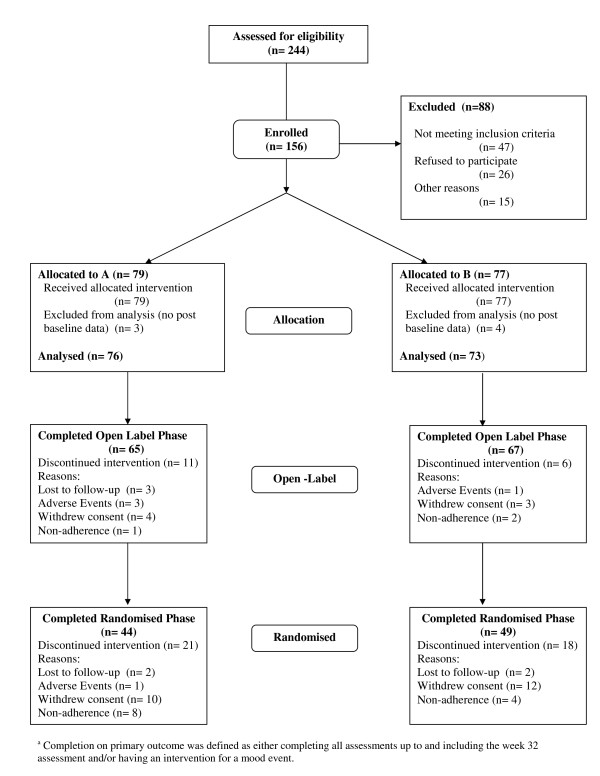
**Consort flowchart for primary and secondary outcome measures**.

One hundred and forty nine individuals meeting DSM-IV-TR criteria for bipolar disorder on a structured clinical interview (MINI-plus) were included in the analysis (see Figure 1). The majority of participants were women, with an average age of 45.8 years (SD = 11.4). Most had bipolar I disorder and the mean duration of time since diagnosis was 10.0 years (SD*= *9.4). Prior suicidality was prevalent in the cohort, and tobacco and alcohol use was also common, but use of other substances of abuse was infrequent.

### Baseline characteristics

The two treatment groups were similar on most demographic (see Table 1), clinical and functioning measures (see Table 2) with the exception that there were significantly more women in the NAC group compared to placebo, χ^2 ^(1) = 8.85, *P = *0.003. Detailed descriptions of the baseline characteristics and the symptom changes in the open-label phase have been previously published [30].

**Table 1:** Descriptive statistics depicting the differences between groups NAC and placebo on demographic and illness characteristics.

Characteristics	Descriptive statistic	Total sample n = 149	NAC n = 76	Placebo n = 73	Test statistic	Value	df	*P*
Age	M(SD)	45.8 (11.4)	47.1 (10.9)	44.4 (11.8)	t-test	1.46	147	.146
Gender %Female	% (n)	67.8 (101)	78.9 (60)	56.2 (41)	χ^2^	8.85	1	.003
								
Diagnosis^a^								
Bipolar I disorder	% (n)	69.6 (103)	73.7 (56)	65.3 (47)	χ^2^	1.24	1	.266
Bipolar II disorder	% (n)	29.7 (44)	25.0 (19)	34.7 (25)				
Bipolar NOS	% (n)	1 (0.7)	1.3 (1)	0.0 (0)				
Suicidality %Yes	% (n)	70.9 (105)	69.7 (53)	72.2. (52)	χ^2^	0.11	1	.739
Age of first symptoms	M(SD)	22.0 (10.6)	22.2 (11.8)	21.9 (9.2)	t-test	0.17	138	.867
Age of diagnosis	M(SD)	35.9 (11.6)	37.4 (12.0)	34.4 (11.2)	t-test	1.58	141	.115
Duration of illness since diagnosis (years)^b^	M(SD)	10.0 (9.4)	9.6 (9.3)	10.5 (9.6)	t-test	-0.74	141	.462
	Mdn	7.0	6.0	7.5				
Number of psychiatric hospitalisations^c^	M(SD)	3.0 (4.4)	3.2 (5.3)	2.8 (3.3)	t-test	-0.31	141	.759
	Mdn	1.0	2.0	1.0				
								
Number of manic episodes >10	% (n)	53.5 (76)	54.8 (40)	52.2 (36)	χ^2^	0.10	1	.754
Number of depressive episodes >10	% (n)	78.3 (112)	80.8 (59)	75.7 (53)	χ^2^	0.55	1	.459
								
Smoker %Yes ^b^	% (n)	37.1 (49)	30.8 (20)	43.3 (29)	χ^2^	2.21	1	.137
Alcohol use %Yes ^b^	% (n)	47.0 (62)	47.7 (31)	46.3 (31)	χ^2^	0.03	1	.870
Alcohol dependence/abuse ^b^	% (n)	14.2 (21)	14.5 (11)	13.9 (10)	χ^2^	0.01	1	.919
Substance use %Yes ^b^	% (n)	3.0 (4)	4.6 (3)	1.5 (1)	χ^2^	1.10	1	.295
Substance dependence/abuse ^b^	% (n)	14.3 (21)	13.3 (10)	15.3 (11)	χ^2^	0.11	1	.736

^a^Chi-square based on two categories (Bipolar I disorder versus other) due to small cell sizes;

^b ^These substances use variables related to status at entry to the maintenance phase of the trial (Week 8). ^c ^Inferential statistics based on logarithmic transformed data (plus constant) because of extreme positive skewness. Untransformed descriptive statistics are reported.

**Table 2:** Differences between groups NAC and placebo on scores at Week 8 (at randomization) in terms of symptoms and functioning.

Characteristics	Descriptive statistic	Total sample n = 149	NAC n = 76	Placebo n = 7 3	Test statistic	Value	df	*P*
Symptoms								
YMRS^a^	M(SD)	1.5 (2.1)	1.7 (2.4)	1.4 (1.9)	t-test	0.37	130	.716
MADRS^b^	M(SD)	12.6 (9.8)	13.1 (9.72)	12.2 (9.9)	t-test	0.47	130	.638
BDRS^a^	M(SD)	10.7 (9.1)	11.3 (8.5)	10.1 (9.7)	t-test	1.20	130	.233
CGI-BP Severity								
Depression^a ^	M(SD)	2.8 (1.2)	2.9 (1.2)	2.8 (1.2)	t-test	0.49	129	.625
Mania^a^	M(SD)	1.3 (0.7)	1.4 (0.7)	1.3 (0.6)	t-test	0.17	129	.620
Overall^a^	M(SD)	2.9 (1.2)	2.9 (1.2)	2.8 (1.2)	t-test	0.29	128	.771
CGI-BP Improvement								
Depression^a^	M(SD)	2.5 (1.2)	2.5 (1.1)	2.6 (1.3)	t-test	-0.17	129	.864
Mania^a^	M(SD)	3.8 (0.7)	3.8 (0.7)	3.9 (0.7)	t-test	-0.63	130	.533
Overall^a^	M(SD)	2.6 (1.2)	2.6 (1.2)	2.7 (1.3)	t-test	-0.30	129	.767
Functioning								
GAF	M(SD)	72.2 (13.7)	71.1 (13.3)	73.3 (14.1)	t-test	-0.92	129	.360
SOFAS	M(SD)	72.1 (13.6)	71.5 (13.0)	72.6 (14.1)	t-test	-0.46	129	.649
LIFE-RIFT	M(SD)	11.2 (3.8)	11.5 (3.9)	10.9 (3.8)	t-test	0.83	130	.410
SLICE-LIFE	M(SD)	16.5 (5.2)	16.6 (5.3)	16.5 (5.0)	t-test	0.17	130	.866
Q-LES-Q	M(SD)	53.4 (10.8)	52.5 (10.6)	54.3 (11.0)	t-test	-0.98	130	.325

YMRS, Young Mania Rating Scale; MADRS, Montgomery-Asberg Depression Rating Scale; BDRS, Bipolar Depression Rating Scale; CGI-BP Clinical Global Impressions - Bipolar Disorder; GAF, Global Assessment of Functioning; SOFAS, Social and Occupational Assessment Scale; Q-LES-Q, Quality of Life Enjoyment and Satisfaction Questionnaire. ^a^Inferential statistics based on logarthmic transformed data (plus constant) because of extreme positive skewness. Untransformed descriptive statistics are reported; ^b^Inferential statistics based on square root transformed data (plus constant) because of positive skewness. Untransformed descriptive statistics are reported.

### Participant flow

Of the 149 participants randomized, 132 (88.6%) completed the open label phase of the study (see Figure 1). There were 121 participants (NAC: 77.6%, n = 59 and placebo: 84.9%, n*= *62) who had at least one follow-up assessment during the maintenance phase of the trial. Participants who did not have data in the maintenance phase of the trial were less likely to have a diagnosis of bipolar I disorder (included group 74.4% (n = 90) had bipolar I disorder versus excluded group 48.1% (n = 13) had bipolar I disorder), χ^2 ^(1) = 7.18, *P = *0.007. For the primary outcome measure, completers of the maintenance phase of the trial were delineated on the basis of either having an event and/or completing all visits. Based on these criteria, 67.8% (n = 101) of the participants had complete data for the primary outcome measure for the maintenance phase of the trial.

There were no significant differences between the two treatment groups with respect to completion rates on the primary outcome variable (completers NAC: 79.1%, n = 47; placebo: 87.1%, n = 54), χ^2 ^(1) = 1.21, *P = *0.271. Participants who did, and did not, have complete data for the primary outcome, were no different with respect to a range of demographic, diagnostic and substance use variables. For the total cohort, completers had significantly lower scores on the LIFE-RIFT (completers *M *= 10.8, SD = 3.9; non-completers *M = *13.0, SD = 3.5, *t*(119) = 2.33, *P *= 0.021) and the SLICE/LIFE (completers *M *= 16.0, SD = 5.1; non-completers *M = *18.7, SD = 4.7, *t*(119) = 2.15, *P *= 0.033) compared to non-completers.

### Time to intervention for a mood episode

**Figure 2:**
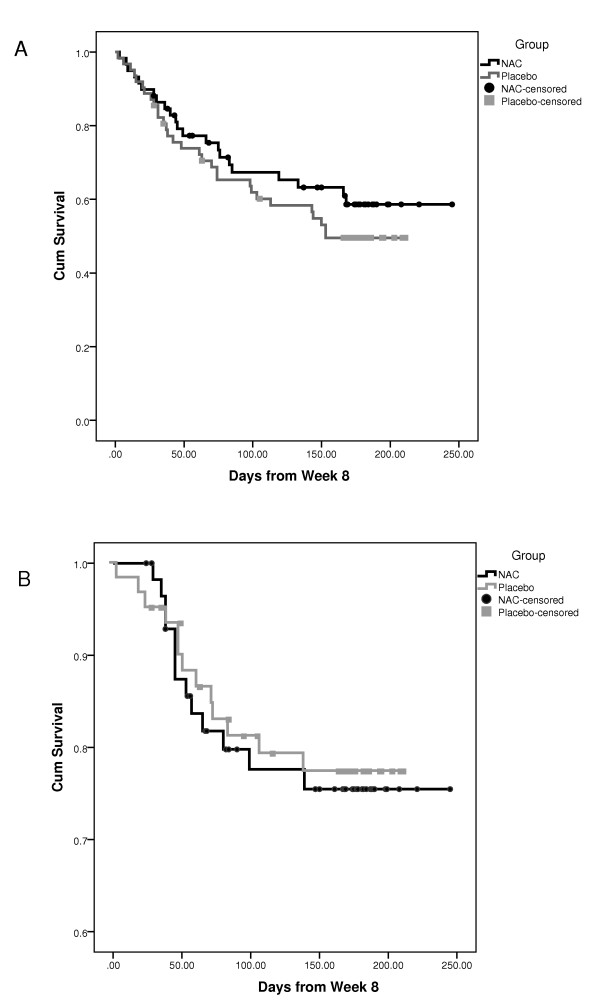
**Survival time depicting time to intervention for mood episode (**A**) and time to depressive episode (**B**)**.

Thirteen interventions for mood events occurred in each of the two groups. There was no significant difference between the two groups in overall event rates, Fisher's exact, *P *= 0.531. The average survival time for the NAC group was not significantly longer in the NAC group (199.9 days, SE*=*11.0, 95%, CI (178.2, 221.5)) than the placebo group (177.5 days, SE = 8.4, 95% CI (161.0, 194.0)), log rank χ^2^(1) = .07, *P *= 0.795 (see Figure 2A

### Time to depressive episode

Twenty-two (37.3%) participants in the NAC and 30 (48.4%) in the placebo group had a depressive episode, Fisher's exact, *P *= 0.147. Although the average survival time (in days) for the NAC group (*M = *170.2, SE = 12.8, 95%, CI (145.1, 195.39)) was longer than for those in the placebo group (*M *= 137.4, SE *= *10.4, 95%, CI (117.0, 157.7)), the difference between groups was not significant, log rank χ^2^(1) = .91, *P *= .341 (see Figure 2B).

### Manic episode

Nine participants had a manic episode during the maintenance phase: two (3.2%) in the placebo group and seven (11.9%) in the NAC group, Fisher's exact, *P *= .070. Given the small number of manic events, survival analysis was not conducted.

### Clinical and functioning measures

No significant group-by-visit interactions were identified in the MMRM models for all clinical, functioning, and QoL measures (see Table 3). Both groups, however, improved significantly over time with respect to depression (MADRS, BDRS) and functioning (SOFAS and GAF) measures. Planned comparisons focusing on group differences in the mean change from week 8 to endpoint (week 32) were non-significant for all measures (see Table 4). Further, there were no significant between group differences on the PGI-I scale at 32 weeks (NAC *M *= 2.3, SD = 1.2*; *placebo *M*= 2 .5*, SD *= 1.4*), P *= 0.451.

**Table 3:** Tests of fixed effects in MMRM for measures of psychopathology and functioning.

Effect	F test	df	*P *value		F test	df	*P *value
Symptoms							
YMRS †				MADRS Total Score†			
Group	2.04	1, 113.4	.156	Group	0.26	1, 121.7	.610
Time	1.45	6, 285.4	.197	Time	3.15	6, 240.6	.005
Group by time	0.99	6, 285.4	.432	Group by time	0.44	6, 240.6	.855
BDRS †							
Group	0.27	1, 124.8	.604				
Time	3.83	6, 245.2	.001				
Group by time	0.49	6, 245.2	.813				
CGI-BP Severity Depression †				CGI-BP Improvement Depression †			
Group	0.03	1, 126.0	.863	Group	0.14	1, 123.6	.709
Time	1.03	6, 197.4	.405	Time	2.43	6, 341.8	.026
Group by time	0.60	6, 197.4	.731	Group by time	0.54	6, 341.8	.777
CGI-BP Severity Mania †				CGI-BP Improvement Mania†			
Group	0.01	1, 116.0	.935	Group	0.05	1, 124.3	.822
Time	0.38	6, 247.2	.893	Time	1.99	6, 303.2	.066
Group by time	0.75	6, 247.2	.614	Group by time	1.32	6, 303.2	.248
CGI-BP Severity Overall †				CGI-BP Improvement Overall†			
Group	0.15	1, 122.7	.704	Group	0.05	1, 125.3	.828
Time	1.31	6, 201.0	.255	Time	3.10	6, 335.6	.006
Group by time	0.47	6, 201.0	.829	Group by time	0.38	6, 335.6	.893
Functioning							
GAF				SOFAS			
Group	1.60	1, 121.9	.209	Group	0.46	1, 121.7	.500
Time	2.90	6, 252.9	.010	Time	4.43	6, 311.8	<.001
Group by time	0.75	6, 252.9	.609	Group by time	0.95	6, 311.8	.460
LIFE-RIFT				SLICE-LIFE			
Group	0.95	1, 121.4	.332	Group	0.12	1, 119.8	.732
Time	1.09	6, 242.0	.370	Time	0.86	6, 275.5	.525
Group by time	0.52	6, 242.0	.794	Group by time	0.62	6, 275.5	.714
QLESQ							
Group	0.53	1, 123.2	.466				
Time	1.99	6, 265.4	.067				
Group by time	0.16	6, 265.4	.987				

YMRS, Young Mania Rating Scale; MADRS, Montgomery-Asberg Depression Rating Scale; BDRS, Bipolar Depression Rating Scale; CGI-BP Clinical Global Impressions - Bipolar Disorder; GAF, Global Assessment of Functioning; SOFAS, Social and Occupational Assessment Scale; LIFE-RIFT, Range of Impaired Functioning Tool; SLICE-LIFE, Streamed Longitudinal Interval Clinical Evaluation of the Longitudinal Interview Follow-Up Evaluation; Q-LES-Q, Quality of Life Enjoyment and Satisfaction Questionnaire. † Inferential statistics based on logarithmic transformed data (plus constant) because of extreme positive skewness.

**Table 4:** Secondary efficacy end points over the 32 weeks.

	Change from Week 8 to Week 32				
Characteristics	NAC	Placebo	t^b^	df	*P*
					
	M (SE)^a^	M (SE)			
Symptoms					
YMRS^†^	0.1 (0.4)	0.4 (0.4)	-0.68	129.7	.500
MADRS^††^	0.6 (1.5)	1.5 (1.5)	-0.42	141.3	.679
BDRS†	1.4 (1.4)	0.7 (1.3)	0.35	139.0	.726
CGI-BP - Severity^†^					
Depression	-0.1 (0.2)	-0.3 (0.2)	0.5	186.5	.615
Mania	0.1 (0.1)	-0.1 (0.1)	0.22	160.7	.826
Overall	0.1 (0.2)	-0.2 (0.2)	0.46	167.4	.646
CGI-BP - Improvement					
Depression	0.1 (0.3)	0.2 (0.3)	-0.24	105.6	.814
Mania ^C^	-0.2 (0.2)	0.0 (0.2)	-1.23	67.6	.222
Overall	0.1 (0.3)	0.3 (0.3)	-0.39	93.4	.695
Functioning					
GAF	-3.2 (1.8)	-4.4 (1.7)	0.54	136.7	.593
SOFAS	-3.6 (1.8)	-4.5 (1.7)	0.36	165.0	.719
LIFE-RIFT	0.5 (0.5)	1.1 (0.5)	-0.76	100.5	.449
SLICE-LIFE	0.2 (0.8)	1.2 (0.8)	-0.89	107.4	.376
QLESQ	-1.7 (1.8)	-2.0 (1.7)	0.14	120.5	.890

YMRS, Young Mania Rating Scale; MADRS, Montgomery-Asberg Depression Rating Scale; BDRS, Bipolar Depression Rating Scale; CGI-BP Clinical Global Impressions - Bipolar Disorder; GAF, Global Assessment of Functioning ; SOFAS, Social and Occupational Assessment Scale; LIFE-RIFT, Range of Impaired Functioning Tool; SLICE-LIFE, Streamed Longitudinal Interval Clinical Evaluation for the Longitudinal Interview Follow-Up Evaluation; Q-LES-Q, Quality of Life Enjoyment and Satisfaction Questionnaire. ^a^Least squares mean (standard error) derived from MMRM; ^b ^Planned comparisons from the MMRM testing the difference in Week 8 to Week 32 change between A and B groups; ^c ^Note that the estimate here is derived without investigator in the model, will have to re-run; † Inferential statistics based on logarithmic transformed data (plus constant) because of extreme positive skewness. Untransformed descriptive statistics are reported; †† Inferential statistics based on square root transformation because of positive skewness. Untransformed descriptive statistics are reported.

### Subgroup analyses

The above analyses were also undertaken for the subgroup of participants who did not respond to NAC in the open label phase of the trial. Non-response was defined as a MADRS score >7 at the end of the open label phase. The findings from these supplementary analyses did not differ from the outcomes of the main analyses.

## Discussion

**Figure 3:**
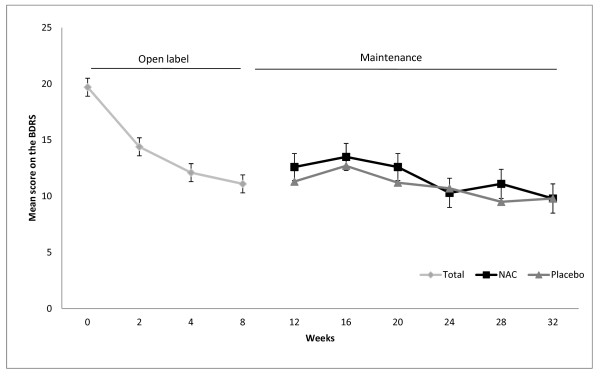
**Mean estimates (± SE) for BDRS scores in the NAC and placebo groups over the open label and maintenance phases of the trial**. BRDS, Bipolar Depression Rating Scale; NAC, N-acetyl cysteine.

Although there was a robust decrease in symptoms in the open-label phase of the trial using 2 grams daily of NAC, there were no significant changes in clinical, functioning and QoL measures in the maintenance phase (see Figure 3 for the changes in mean estimates for the BDRS over both the open-label and maintenance phase of the trial). The improvements in depressive symptoms reached a plateau in the open-label phase and symptoms changed little from this very low base in the randomized phase, such that between group differences did not emerge.

These results are not concordant with our previous data examining NAC in bipolar disorder [31]. In a 24-week double blind placebo controlled trial (n = 75) adjunctive NAC was trialled in participants with either bipolar I or II disorder [32]. In this study NAC significantly improved clinical outcomes, particularly depression, quality of life and measures of functioning, with large effect sizes in almost all domains. In that trial, between group differences at endpoint were no longer evident four weeks after treatment discontinuation. In addition, benefits of NAC in other psychiatric disorders, including a large clinical trial of schizophrenia [8], and small scale clinical papers in obsessive-compulsive disorder [33] and compulsive disorders [9,33,34], pathological gambling [35] and cocaine dependence [36] have been reported.

NAC reverses models of glutathione depletion, increasing peripheral [37] and brain glutathione [38]. There appears to be reduced neurogenesis in mood disorders, and antidepressants and mood stabilizers enhance neurogenesis [39]. It is noteworthy in this regard that NAC enhances neurogenesis of neuronal stem cells. NAC promotes neuronal survival after injury and has anti-inflammatory effects. In the forced swim test, NAC results in a significant decrease in immobility. A neuroprotective effect of NAC has been suggested by effects in a variety of neurodegenerative disease models and lastly NAC appears to reverse mitochondrial dysfunction, which is concordant with the emerging literature on the role of mitochondria in bipolar disorder.

The characteristics of this study need to be mentioned in order to contextualize these results. The multi-center, randomized, placebo controlled design provides an appropriate forum for evaluation of efficacy in the continuation phase. The sample size, while ample for the secondary outcome measures, was marginal for the ability to evaluate recurrence, which only occurred in a subset of participants. A larger sample size and a longer length of the randomized phase, concordant with similar recent designs, would have increased the signal to noise ratio in this regard. Other studies have used responder analysis to determine benefits during the maintenance phase; these trials only include those who respond in the open-label phase for analysis of the maintenance phase. While not an *a priori *analysis, this was investigated in the current study with no change in the outcomes reported (data not shown). The absence of significant restrictions on comorbid diagnosis reflects the extent of comorbidity in the disorder; similarly, the absence of restrictions regarding concomitant therapy also enhances the generalizability of the data, as does the inclusion of bipolar I-, II- and NOS - disorder participants.

## Conclusions

In conclusion, there was a substantial decrease in symptoms during the eight-week open label phase. During the subsequent double blind phase, there was a very low baseline reflecting improvement across all symptomatic measures, and this did not change significantly in either group. As a consequence, between group differences could not emerge on recurrence, clinical functioning and QoL measures. This lack of a workable symptomatic substrate for the emergence of a signal in the double blind phase suggests that this study could possibly be seen as a failed rather than negative study. As such, the formal maintenance efficacy of NAC in bipolar disorder remains an open question.

## Abbreviations

BDRS: Bipolar Depression Rating Scale; CGI: Clinical Global Improvement; GAF: Global Assessment of Functioning Scale; LIFE RIFT: Range of Impaired Functioning Tool; MADRS: Montgomery Åsberg Depression Rating Scale; MMRM: mixed-effects model repeated measures approach; NAC: N-acetyl cysteine; PGI Patient Global Impression; Q-LES-Q: Quality of Life Enjoyment and Satisfaction Questionnaire; QoL: Quality of life; SLICE-LIFE: Streamed Longitudinal Interview Clinical Evaluation from the Longitudinal Interview Follow-up Evaluation; SOFAS: Social and Occupational Functioning Assessment Scale; YMRS: Young Mania Rating Scale.

## Competing interests

MB has received grant support from NIH, Simons Autism Foundation, Cancer Council of Victoria, Stanley Medical Research Foundation, MBF, NHMRC, Beyond Blue, Geelong Medical Research Foundation, Bristol Myers Squibb, Eli Lilly, GlaxoSmithKline, Organon, Novartis, Mayne Pharma and Servier. MB has been a speaker for Astra Zeneca, Bristol Myers Squibb, Eli Lilly, GlaxoSmithKline, Janssen Cilag, Lundbeck, Merck, Pfizer, Sanofi Synthelabo, Servier, Solvay and Wyeth, and served as a consultant to Astra Zeneca, Bristol Myers Squibb, Eli Lilly, GlaxoSmithKline, Janssen Cilag, Lundbeck and Servier. AIB is a shareholder in Prana Biotechnology Ltd, Cogstate Ltd, and Eucalyptus Pty Ltd. MB and AIB are co-inventors on two provisional patents regarding the use of NAC and related compounds for psychiatric indications, assigned to the Mental Health Research Institute. OMD has received grant support from Simons Autism Foundation, Stanley Medical Research Institute, Lilly, NHMRC and an ASBD/Servier grant. GM has received research support from AstraZeneca, Eli Lilly, Organon, Pfizer, Servier, and Wyeth; has been a speaker for AstraZeneca, Eli Lilly, Janssen Cilag, Lundbeck, Pfizer, Ranbaxy, Servier, and Wyeth; and has been a consultant for AstraZeneca, Eli Lilly, Janssen Cilag, Lundbeck, and Servier. CG has received grant/research support from CNPq, FIPEe HCPA, and FAPERGS, and has been a speaker/advisory for AstraZeneca, Lundbeck, Pfizer, Actelion. **IS, SC, CA, KH, KK, KM, SJ **have no competing interests to disclose.

## Authors' contributions

 MB was involved in the initial drafting and development of the manuscript, protocol development and study oversight. OD was responsible for managing the conduct of the study. KK also assisted in managing the study. SC was responsible for leading the statistical analysis for the study. FK was a lead investigator at the Brazil site and oversaw the project at that site. CG, BF, SJ, KH, KM, CA, IS, HC were all responsible for recruitment of participants and participant interviews. AB and SD were involved in the development of the protocol and study design. GM was the lead investigator at the Sydney site and oversaw the project at that site. All authors made a substantial contribution to drafting and developing the final manuscript. All authors read and approved the final manuscript.

## Pre-publication history

The pre-publication history for this paper can be accessed here:

http://www.biomedcentral.com/1741-7015/10/91/prepub

## Supplementary Material

Additional file 1 **Timetable for assessments during the open label and maintenance phases of the trial**. This table provides an outline of the trial schedule and timing of rating scale and associated trial interview delivery. Click here for file
