# Supplementary material for: Maintenance N-acetyl cysteine treatment for bipolar disorder: A double-blind randomized placebo controlled trial
Source: BMC Med. 2012 Aug 14;10:91. doi: 10.1186/1741-7015-10-91 (PMC3482580; doi:10.1186/1741-7015-10-91)
Supplement: Additional file 1 — Timetable for assessments during the open label and maintenance phases of the trial. This table provides an outline of the trial schedule and timing of rating scale and associated trial interview delivery. [file 1741-7015-10-91-S1.DOCX]

**Additional File 1**

Timetable for assessments during the open label and maintenance phases of the trial

|  |  |  | Open Label NAC Treatment | | | **Randomised Placebo-Controlled Phase** | | | | | |
| --- | --- | --- | --- | --- | --- | --- | --- | --- | --- | --- | --- |
|  | *Screen* | *Baseline* |  | | |  | | | | | |
| **WEEK No.** | **Week 0** | **Week 0** | **Week 2** | **Week 4** | **Week 8** | **Week 12** | **Week 16** | **Week 20** | **Week 24** | **Week 28** | **Week 32** |
| Informed Consent | X | - | - | - | - | - | - | - | - | - | - |
| MINI PLUS Interview | X | - | - | - | - | - | - | - | - | - | - |
| Pregnancy Test | X | X | - | - | - | - | - | - | - | - | - |
| Entrance Criteria | X | X | - | - | - | - | - | - | - | - | - |
| Family History | X | X | - | - | - | - | - | - | - | - | - |
| Psychiatric History | X | X | - | - | - | - | - | - | - | - | - |
| Habits |  | X | X | X | X | X | X | X | X | X | X |
| T.I.M.E. | - | - | X | X | X | X | X | X | X | X | X |
| Key/Adverse Events | - | - | X | X | X | X | X | X | X | X | X |
| AUDIT | - | - | X | X | X | X | X | X | X | X | X |
| Q-LES-Q (short form) | - | X | X | X | X | X | X | X | X | X | X |
| BDRS | - | X | X | X | X | X | X | X | X | X | X |
| MADRS | - | X | X | X | X | X | X | X | X | X | X |
| YMRS | - | X | X | X | X | X | X | X | X | X | X |
| SLICE/LIFE | - | X | X | X | X | X | X | X | X | X | X |
| LIFE/RIFT | - | X | X | X | X | X | X | X | X | X | X |
| GAF | - | X | X | X | X | X | X | X | X | X | X |
| SOFAS | - | X | X | X | X | X | X | X | X | X | X |
| CGI-BP | - | - | X | X | X | X | X | X | X | X | X |
| CGI-S | - | X | X | X | X | X | X | X | X | X | X |
| PGI | - | X | X | X | X | X | X | X | X | X | X |

MINI PLUS-BP Interview – Mini International Neuropsychiatric Interview for Bipolar Disorder

T.I.M.E – Time to Invention for Mood Episode

Q-LES-Q (short form) – Quality of Life Enjoyment and Satisfaction Questionnaire – Short Form

*BDRS – Bipolar Depression Rating Scale*

MADRS– Montgomery-Asberg Depression Rating Scale

YMRS – Young Mania Rating Scale

SLICE/LIFE - Streamlined Longitudinal Interview Clinical Evaluation for the Longitudinal Interval Follow-up Evaluation

LIFE-RIFT - Longitudinal Interval Follow-up Evaluation – Range of Impairment Functioning Tool

GAF – Global Assessment of Functioning – Bipolar Disorder

SOFAS – Social and Occupational Functioning Assessment Scale

CGI-S adapted for Alcohol and Drug Use – Clinical Global Impression of Alcohol and Drug Use

CGI-BP – Clinical Global Impression – Bipolar Disorder

PGI - Patient Global Impression
